# A Comparative Analysis of In-Hospital Mortality per Disease Groups in Germany Before and During the COVID-19 Pandemic From 2016 to 2020

**DOI:** 10.1001/jamanetworkopen.2021.48649

**Published:** 2022-02-15

**Authors:** Sebastian König, Vincent Pellissier, Sven Hohenstein, Johannes Leiner, Gerhard Hindricks, Andreas Meier-Hellmann, Ralf Kuhlen, Andreas Bollmann

**Affiliations:** 1 Department of Electrophysiology, Heart Center Leipzig at the University of Leipzig, Leipzig, Germany; 2 Leipzig Heart Institute, Leipzig, Germany; 3 Helios Hospitals, Berlin, Germany; 4 Helios Health, Berlin, Germany

## Abstract

**Importance:**

Throughout the ongoing SARS-CoV-2 pandemic, it has been critical to understand not only the viral disease itself but also its implications for the overall health care system. Reports about excess mortality in this regard have mostly focused on overall death counts during specific pandemic phases.

**Objective:**

To investigate hospitalization rates and compare in-hospital mortality rates with absolute mortality incidences across a broad spectrum of diseases, comparing 2020 data with those of prepandemic years.

**Design, Setting, and Participants:**

Retrospective, cross-sectional, multicentric analysis of administrative data from 5 821 757 inpatients admitted from January 1, 2016, to December 31, 2020, to 87 German Helios primary to tertiary care hospitals.

**Exposures:**

Exposure to SARS-CoV-2.

**Main Outcomes and Measures:**

Administrative data were analyzed from January 1, 2016, to March 31, 2021, as a consecutive sample for all inpatients. Disease groups were defined according to *International Statistical Classification of Diseases and Related Health Problems, 10th Revision* (*ICD-10*; German modification) encoded main discharge diagnoses. Incidence rate ratios (IRRs) for hospital admissions and hospital mortality counts, as well as relative mortality risks (RMRs) comparing 2016-2019 with 2020 (exposure to the SARS-CoV-2 pandemic), were calculated with Poisson regression with log-link function.

**Results:**

Data were examined for 5 821 757 inpatients (mean [SD] age, 56.4 [25.3] years; 51.5% women), including 125 807 in-hospital deaths. Incidence rate ratios for hospital admissions were associated with a significant reduction for all investigated disease groups (IRR, 0.82; 95% CI, 0.79-0.86; *P* < .001). After adjusting for age, sex, the Elixhauser Comorbidity Index score, and SARS-CoV-2 infections, RMRs were associated with an increase in infectious diseases (RMR, 1.28; 95% CI, 1.21-1.34; *P* < .001), musculoskeletal diseases (RMR, 1.19; 95% CI, 1.04-1.36; *P* = .009), and respiratory diseases (RMR, 1.09; 95% CI, 1.05-1.14; *P* < .001) but not for the total cohort (RMR, 1.00; 95% CI, 0.99-1.02; *P* = .66). Regarding in-hospital mortality, IRR was associated with an increase within the *ICD-10* chapter of respiratory diseases (IRR, 1.28; 95% CI, 1.13-1.46; *P* < .001) in comparing 2020 with 2016-2019, in contrast to being associated with a reduction in IRRs for the overall cohort and several other subgroups. After exclusion of patients with SARS-CoV-2 infections, IRRs were associated with a reduction in absolute in-hospital mortality for the overall cohort (IRR, 0.78; 95% CI, 0.72-0.84; *P* < .001) and the subgroup of respiratory diseases (IRR, 0.83; 95% CI, 0.74-0.92; *P* < .001).

**Conclusions and Relevance:**

This cross-sectional study of inpatients from a multicentric German database suggests that absolute in-hospital mortality for 2020 across disease groups was not higher compared with previous years. Higher IRRs of in-hospital deaths observed in patients with respiratory diseases were likely associated with individuals with SARS-CoV-2 infections.

## Key Points

**Question:**

What association does the COVID-19 pandemic have with inpatient care in different disease groups?

**Findings:**

In this cross-sectional study of 5 821 757 inpatients’ administrative data, hospital admission rates were lower for all investigated disease groups in 2020 compared with previous years. Despite higher relative mortality in some subgroups, a higher absolute incidence of in-hospital deaths was observed only for respiratory diseases, which was associated with patients with SARS-CoV-2 infections.

**Meaning:**

In 2020, a higher absolute in-hospital mortality was observed only in patients with respiratory diseases, but not in other disease groups or overall.

## Teaser

This cross-sectional study uses administrative data from 87 German hospitals to examine hospitalization and in-hospital mortality rates across a broad spectrum of diseases, comparing 2020 data with those of prepandemic years.

## Introduction

The COVID-19 pandemic and subsequent consequences led to profound changes in health care worldwide in 2020. Excess all-cause mortality was reported during incidence peaks of infections for several countries.^1,2,3,4,5,6,7^ Moreover, significant reductions in inpatient hospital admission rates were observed in parallel to the pandemic waves for cardiovascular, respiratory, and intestinal diseases.^8,9,10,11^ Especially in studies investigating patient treatment pathways for cardiac diseases, those hospitalization deficits were accompanied by an increased relative in-hospital mortality rate in some cohorts.^12,13,14^ Whether numbers of absolute deaths were also increased in those or other disease groups is unclear because of lacking data but must be considered as 1 step toward adequately interpreting the implications of the pandemic on the health care system. Therefore, the aim of this study was to investigate hospitalization rates and compare relative in-hospital mortality rates with absolute mortality incidences across a broad spectrum of disease groups, comparing data from 2020 with those of the prepandemic years of 2016-2019 to better understand inpatient care pathways.

## Methods

In this retrospective, cross-sectional analysis, we analyzed administrative data on inpatients from 87 Helios hospitals in Germany from January 1, 2016, to March 31, 2021. Information about race and ethnicity was not available in the data set used for this analysis. All completed cases of inpatients admitted within January to December of one year and discharged up to March 31 the following year were assigned to the corresponding year of admission and studied without further selection criteria. Repeated hospitalizations per patient were possible; all analyses were performed according to patient cases and not individual patients owing to data structure. Cases not meeting the mentioned criteria with respect to admission and discharge date were excluded from further analyses. Patients admitted from 2016-2019 were grouped and compared with those from 2020. Cause-specific hospitalizations were defined according to the encoded primary diagnosis at hospital discharge, based on the *International Statistical Classification of Diseases and Related Health Problems, 10th Revision *(*ICD-10*; German modification)*,* using the predefined *ICD-10* disease categories (eTable 1 in the Supplement). *ICD-10* chapters with a maximum number of deaths per year below 100 were excluded. Patients with laboratory-confirmed SARS-CoV-2 infection were identified via the specific *ICD-10* code (U07.1) irrespective of the mode of infection (community acquired vs nosocomial). Relevant comorbidities defined by the Elixhauser Comorbidity Index score were identified from encoded secondary diagnoses at hospital discharge according to previous publications.^15,16^ Detailed information about *ICD-10* codes used is listed in eTable 2 in the Supplement. In-hospital mortality was defined via the type of hospital discharge and has been assigned as an event to the admission date. For mortality analysis, all case patients discharged as hospital transfers to other acute care hospitals or discharged without a specification of discharge type were excluded (3.4% of all case patients). Patients’ data were stored in an anonymized form and data use was approved by the Helios Kliniken GmbH data protection authority and the local ethics committee. Considering the retrospective analysis of anonymized administrative clinical routine data, individual informed consent was not obtained in accordance with the national legislation and the institutional data protection authorities. The study followed the Strengthening the Reporting of Observational Studies in Epidemiology (STROBE) reporting guideline for cross-sectional investigations.

### Statistical Analysis

Administrative data were extracted from QlikView version 12.30 (QlikTech), and statistical analyses were executed with R version 4.0.2 (R Foundation for Statistical Computing).^17^ Incidence rates for daily admissions within disease groups were calculated by dividing the number of cumulative admissions by the number of days for each period. Mortality rates per 100 000 admissions stratified by *ICD-10* chapter were computed, with the total number of admissions per chapter as the number at risk. Incidence rate ratios (IRRs) comparing 2016-2019 with 2020 (exposure to the SARS-CoV-2 pandemic) were calculated with mixed-effect Poisson regression with log-link function on daily admission count and daily mortality count data, with hospitals as random factors (random intercept and slope). No offset was added to the regression analysis. To prevent overfitting zeroes (zero inflation), excess zeroes were modeled by adding the hospital volume as a zero-inflation term. Because of this, hospitals without an admission count for every year (owing to new acquisitions by the hospital network) were removed to avoid introducing artificial zeroes. For the same reason, hospitals without any admission were removed before analysis. Relative mortality risks (RMRs) were computed with mixed Poisson regression with log-link function on individual mortality data (1/0) with hospitals as random factors (random intercept). Incidence rate ratios and RMRs were calculated with and without correction for age, sex, Elixhauser Comorbidity Index score, and SARS-CoV-2 infection status. In the case of missing data and therefore count values of zero (eg, owing to inconsistent data availability), the association with the estimated effects was lower because of the shrinkage to the population mean. For all Poisson models, we performed tests for overdispersion by calculating the sum of squared Pearson residuals and compared them with the residual *df*, showing that overdispersion was not present. All analyses were also performed by subdividing the reference period into 2 cohorts (2016/2017 and 2018/2019), computing IRRs and RMRs for each group compared with the 2020 cohort. We report IRRs and RMRs together with 95% CIs and *P* values. Smooth curves used to illustrate the temporal evolution of weekly admission rates, as well as deaths, were fitted via locally estimated scatterplot smoothing (degree of smoothing α = .25), presented including a 95% CI. For all tests we applied a 2-tailed 5% error criterion for significance.

## Results

We analyzed cases for 5 821 757 inpatients (mean [SD] age, 56.4 [25.3] years; 51.5% women, 48.5% men; 4 793 836 cases from 2016-2019, with an average of 1 198 459 hospital admissions per year; 1 027 921 cases in 2020) and 125 807 in-hospital deaths (100 815 deaths from 2016-2019, with an average of 25 204 in-hospital deaths per year; 24 992 deaths in 2020).

Incidence rate ratios for averaged daily admission numbers were associated with a significant reduction both overall (IRR, 0.82; 95% CI, 0.79-0.86; *P* < .001) and for all investigated *ICD-10* chapters (Table 1). Locally estimated scatterplot smoothing curves to illustrate weekly admission numbers overall are presented in the Figure. The overall hospitalization deficit was mainly due to a reduction of inpatient cases in the intervals corresponding to governmental restrictions related to the SARS-CoV-2 pandemic (March to May and October to December).^18^ Unadjusted RMRs were associated with a significant increase in 2020 overall (RMR, 1.15; 95% CI, 1.13-1.16; *P* < .001) and for the several subgroups of *ICD-10* chapters (Table 2). When adjusted for age, sex, and the Elixhauser Comorbidity Index score, RMRs remained associated with an increase for the overall population (RMR, 1.09; 95% CI, 1.08-1.11; *P* < .001), as well as for the group of infectious and parasitic diseases (RMR, 1.32; 95% CI, 1.25-1.39; *P* < .001), musculoskeletal diseases (RMR, 1.30; 95% CI, 1.14-1.47; *P* < .001), diseases of the nervous system (RMR, 1.14; 95% CI, 1.03-1.27; *P* = .02), respiratory diseases (RMR, 1.52; 95% CI, 1.47-1.58; *P* < .001), and other diseases (RMR, 1.06; 95% CI, 1.01-1.11; *P* = .02). Lower RMR was observed for the disease group of the genitourinary system (RMR, 0.92; 95% CI, 0.86-0.98; *P* = .007). When SARS-CoV-2 infections were also adjusted for as a covariate, RMR was associated with an increase only for infectious and parasitic diseases (RMR, 1.28; 95% CI, 1.21-1.34; *P* < .001), musculoskeletal diseases (RMR, 1.19; 95% CI, 1.04-1.36; *P* = .009), and respiratory diseases (RMR, 1.09; 95% CI, 1.05-1.14; *P* < .001) but not for the total sum of inpatient cases irrespective of disease group (RMR, 1.00; 95% CI, 0.99-1.02; *P* = .66). Results for unadjusted and adjusted RMR analyses are presented in Table 2.

**Table 1.** Incidence Rate Ratios (IRRs) for Hospital Admissions Comparing the Average of 2016-2019 With That of 2020

*ICD-10* chapter	Hospital admissions	
	IRR (95% CI)^a^	*P* value^b^
Total cohort	0.82 (0.79-0.86)	<.001
Certain infectious and parasitic diseases	0.63 (0.59-0.67)	<.001
Neoplasms	0.83 (0.77-0.89)	<.001
Diseases of the blood and blood-forming organs and certain disorders involving the immune system	0.88 (0.83-0.93)	<.001
Endocrine, nutritional, and metabolic disorders	0.83 (0.77-0.90)	<.001
Mental, behavioral, and neurodevelopmental disorders	0.76 (0.70-0.83)	<.001
Diseases of the nervous system	0.66 (0.58-0.76)	<.001
Diseases of the circulatory/cardiovascular system	0.76 (0.69-0.83)	<.001
Diseases of the respiratory system	0.77 (0.71-0.83)	<.001
Diseases of the digestive system	0.80 (0.73-0.87)	<.001
Diseases of the skin and subcutaneous tissue	0.77 (0.72-0.82)	<.001
Diseases of the musculoskeletal system and connective tissue	0.79 (0.73-0.85)	<.001
Diseases of the genitourinary system	0.85 (0.79-0.91)	<.001
Other diseases	0.82 (0.78-0.87)	<.001

Abbreviation: *ICD-10, International Statistical Classification of Diseases and Related Health Problems, 10th Revision* (German modification).

^a^ Incidence rate ratios for hospital admissions across *ICD-10* chapters comparing the average of 2016-2019 with that of 2020.

^b^ All *P* values are significant.

**Figure.**
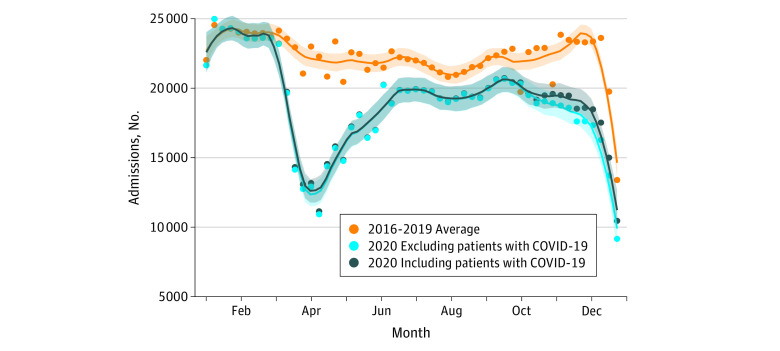
Locally Estimated Scatterplot Smoothing Curves for Weekly Admissions Comparing the Average of 2016-2019 With That of 2020, Stratified for SARS-CoV-2 Cases Shaded areas indicate 95% CIs.

**Table 2.** Unadjusted and Adjusted Relative Mortality Risks (RMRs) per *ICD-10* Chapter Comparing 2016-2019 With 2020

*ICD-10* chapter	Unadjusted RMRs		RMRs adjusted for age, sex, and Elixhauser Comorbidity Index score		RMRs adjusted for age, sex, Elixhauser Comorbidity Index score, and SARS-CoV-2 infections	
	RMR (95% CI)	*P* value	RMR (95% CI)	*P* value	RMR (95% CI)	*P* value
Total cohort	1.15 (1.13-1.16)	<.001^a^	1.09 (1.08-1.11)	<.001^a^	1.00 (0.99-1.02)	.66
Certain infectious and parasitic diseases	1.40 (1.33-1.47)	<.001^a^	1.32 (1.25-1.39)	<.001^a^	1.28 (1.21-1.34)	<.001^a^
Neoplasms	1.01 (0.98-1.04)	.63	0.99 (0.96-1.03)	.73	0.98 (0.95-1.01)	.12
Diseases of the blood and blood-forming organs and certain disorders involving the immune system	1.11 (0.92-1.34)	.29	1.09 (0.90-1.31)	.39	1.03 (0.85-1.25)	.77
Endocrine, nutritional, and metabolic disorders	1.02 (0.93-1.11)	.73	0.98 (0.90-1.07)	.72	0.95 (0.87-1.04)	.25
Mental, behavioral, and neurodevelopmental disorders	1.11 (0.93-1.32)	.25	1.12 (0.93-1.33)	.23	1.11 (0.93-1.32)	.27
Diseases of the nervous system	1.20 (1.08-1.34)	<.001^a^	1.14 (1.03-1.27)	.02^a^	1.11 (0.99-1.24)	.06
Diseases of the circulatory/cardiovascular system	1.05 (1.02-1.07)	.001^a^	1.01 (0.98-1.04)	.52	0.99 (0.96-1.02)	.40
Diseases of the respiratory system	1.70 (1.64-1.76)	<.001^a^	1.52 (1.47-1.58)	<.001^a^	1.09 (1.05-1.14)	<.001^a^
Diseases of the digestive system	1.03 (0.98-1.08)	.21	0.95 (0.91-1.00)	.06	0.93 (0.89-0.98)	.003^a^
Diseases of the skin and subcutaneous tissue	1.05 (0.87-1.26)	.64	1.02 (0.85-1.23)	.82	0.97 (0.80-1.18)	.75
Diseases of the musculoskeletal system and connective tissue	1.31 (1.16-1.49)	<.001^a^	1.30 (1.14-1.47)	<.001^a^	1.19 (1.04-1.36)	.009^a^
Diseases of the genitourinary system	1.00 (0.94-1.06)	.98	0.92 (0.86-0.98)	.007^a^	0.88 (0.83-0.94)	<.001^a^
Other diseases	1.10 (1.05-1.15)	<.001^a^	1.06 (1.01-1.11)	.02^a^	0.99 (0.95-1.04)	.77

Abbreviation: *ICD-10, International Statistical Classification of Diseases and Related Health Problems, 10th Revision* (German modification).

^a^ Significant *P* value.

When IRRs for averaged absolute death counts per day were calculated, a higher IRR was found only for the subgroup of respiratory diseases (IRR, 1.28; 95% CI, 1.13-1.46; *P* < .001) and not for the total cohort (IRR, 0.86; 95% CI, 0.80-0.93; *P* < .001) or other subgroups in which a lower or unchanged mortality based on IRRs was found (Table 3). When the most frequently used codes within the *ICD-10* chapter of respiratory diseases were further specified, mortality associated with a main diagnosis of *ICD-10* code J12 (viral pneumonia, not classified elsewhere) was associated with a marked IRR increase (IRR, 120.1; 95% CI, 31.7-455.5; *P* < .001), whereas there was no association with increased IRRs for the next most frequently used *ICD-10* codes in this group. After exclusion of patients with SARS-CoV-2 infection, IRRs were significantly associated with decreased IRRs for the total cohort (IRR, 0.78; 95% CI, 0.72-0.84; *P* < .001), infectious and parasitic diseases (IRR, 0.72; 95% CI, 0.59-0.87; *P* < .001), neoplasms (IRR, 0.70; 95% CI, 0.61-0.82; *P* < .001), endocrine diseases (IRR, 0.85; 95% CI, 0.72-1.00; *P* = .046), nervous diseases (IRR, 0.80; 95% CI, 0.65-0.99; *P* = .04), circulatory/cardiovascular diseases (IRR, 0.82; 95% CI, 0.76-0.89; *P* < .001), respiratory diseases (IRR, 0.83; 95% CI, 0.74-0.92; *P* < .001), and diseases of the digestive system (IRR, 0.77; 95% CI, 0.68-0.87; *P* < .001). No association with increased IRR was found for the *ICD* group of viral pneumonia (*ICD-10* code J12) after the exclusion of patients with SARS-CoV-2. Detailed results regarding IRRs for absolute in-hospital death numbers are presented in Table 3. For patients with emergency hospital admission only, comparable results were found for the overall population and patients within the single *ICD-10* chapters. Subdividing the reference cohort into a 2016/2017 and a 2018/2019 cohort showed no changes for the IRR analyses. There were minor changes regarding the RMR analyses within the disease groups of nervous, musculoskeletal, and genitourinary diseases, as well as neoplasms. Detailed results of those RMR analyses are provided in eTables 3 and 4 in the Supplement.

**Table 3.** Incidence Rate Ratios (IRRs) for Daily In-Hospital Deaths Comparing 2016-2019 With 2020

*ICD-10* chapter	Averaged daily in-hospital deaths, mean			IRRs including patients with SARS-CoV-2 infections		IRRs excluding patients with SARS-CoV-2 infections	
	2016-2019	2020^a^	2020^b^	IRR (95% CI)	*P* value	IRR (95% CI)	*P* value
Total cohort	69.24	68.66	61.65	0.86 (0.80-0.93)	<.001^c^	0.78 (0.72-0.84)	<.001^c^
Certain infectious and parasitic diseases	5.42	5.13	4.84	0.75 (0.62-0.91)	.003^c^	0.72 (0.59-0.87)	<.001^c^
Neoplasms	14.73	13.79	13.46	0.71 (0.61-0.83)	<.001^c^	0.70 (0.61-0.82)	<.001^c^
Diseases of the blood and blood-forming organs and certain disorders involving the immune system	0.38	0.37	0.35	1.04 (0.84-1.18)	.72	0.99 (0.80-1.23)	.92
Endocrine, nutritional, and metabolic disorders	1.95	1.71	1.63	0.89 (0.75-1.04)	.13	0.85 (0.72-1.00)	.046^c^
Mental, behavioral, and neurodevelopmental disorders	0.45	0.42	0.41	0.89 (0.64-1.23)	.47	0.86 (0.62-1.19)	.36
Diseases of the nervous system	1.25	1.15	1.10	0.78 (0.61-0.98)	.03^c^	0.80 (0.65-0.99)	.04^c^
Diseases of the circulatory/cardiovascular system	19.00	17.71	17.21	0.85 (0.78-0.91)	<.001^c^	0.82 (0.76-0.89)	<.001^c^
Diseases of the respiratory system	8.59	12.16	7.32	1.28 (1.13-1.46)	<.001^c^	0.83 (0.74-0.92)	<.001^c^
Diseases of the digestive system	6.25	5.63	5.44	0.80 (0.71-0.90)	<.001^c^	0.77 (0.68-0.87)	<.001^c^
Diseases of the skin and subcutaneous tissue	0.45	0.36	0.34	0.85 (0.67-1.06)	.15	0.80 (0.64-1.01)	.06
Diseases of the musculoskeletal system and connective tissue	0.79	0.84	0.77	1.04 (0.89-1.22)	.60	1.97 (0.85-1.10)	.61
Diseases of the genitourinary system	3.64	3.31	3.15	0.92 (0.82-1.04)	.18	0.89 (0.79-1.01)	.07
Other diseases	6.32	6.06	5.61	0.89 (0.79-0.99)	.03^c^	0.82 (0.73-0.91)	<.001^c^

Abbreviation: *ICD-10, International Statistical Classification of Diseases and Related Health Problems*, *10th Revision* (German modification).

^a^ Including patients with SARS-CoV-2 infection.

^b^ Excluding patients with SARS-CoV-2 infection.

^c^ Significant *P* value.

## Discussion

In this cross-sectional study of inpatients from a multicentric German database, we showed higher relative in-hospital mortality rates in 2020 compared with previous years within several disease groups even after adjustment for baseline variables. This was accompanied by lower hospital admission rates in the same period for all investigated *ICD-10* chapters. Absolute death count investigation showed a significantly higher rate in 2020 only within the subgroup of respiratory diseases. The latter may be associated with SARS-CoV-2–related sequelae because not a higher but a lower IRR for in-hospital mortality was found after exclusion of patients with a concomitant SARS-CoV-2 infection. Significantly lower IRRs for mortality were found for the total cohort, and IRRs were lower or unchanged for all other disease groups except respiratory diseases.

To our knowledge, this is the first analysis focusing on changes of in-hospital mortality across a wide spectrum of disease groups during the SARS-CoV-2 pandemic beyond the direct associations of disease prevalence on mortality in patients with COVID-19. Most existing statistics regarding pandemic-associated excess mortality are reporting overall excess deaths (inpatient and outpatient) within specific high-incidence periods.^19,20,21,22^ To our knowledge, there are no comparable investigations regarding in-hospital deaths or a structured evaluation of mortality within different disease groups. However, several studies reported excess mortality including inpatients and outpatients associated with single disease groups. Liu et al^23^ reported a higher cause-specific mortality associated with cardiovascular diseases and diabetes during the first pandemic wave in Wuhan District. In contrast to our analysis, the authors took both inpatient and outpatient mortality data into account for their calculation, which is likely to influence results because reduced rates of emergency service activations and admission rates for acute coronary syndromes, accompanied by a longer time to first medical contact and a significantly higher rate of out-of-hospital cardiac arrests, were observed in other regions.^13,24,25,26^ A postponement of planned interventions, as well as patients’ reluctance to enter the health care system, may be explanations for those findings.^27,28,29^ Sharma et al^30^ described an association of a decreased rate of stroke-related calls to emergency services with excess cerebrovascular deaths within the next 2 weeks and an overall excess mortality rate associated with cerebrovascular disease between March and May 2020 in the United States. Higher overall mortality was also shown in a primary care cohort of patients with cancer in April 2020 in England. Urgent hospital admissions declined by 70% during this first pandemic wave in this group of patients.^31^ In contrast, mortality rates within hospitalized patients with cancer who were receiving chemotherapy were unchanged in spring 2020 according to an analysis from a single tertiary center.^32^ However, all of those analyses focused on specific intervals within 2020, which hinders direct comparison because our study examined hospitalization and death rates throughout the whole year.

There are several possible explanations for the above-mentioned findings. First, the higher relative risk for in-hospital mortality in some subgroups may be a consequence of patient selection, with patients with only the most severe disease presenting to the hospital. When cardiovascular cohorts are examined, an association with increased disease severity with respect to worsened symptoms has been found in several studies, even though this has not always been associated with concomitant increased mortality.^14,33,34^ In addition, the above-mentioned prolonged time to first medical contact that has been demonstrated for several disease entities could have actually led to an increase in the proportion of patients with severe disease owing to delayed treatment. Second, excess mortality may have been limited to periods with particularly high incidences of SARS-CoV-2 infections and have been offset by the periods in between in the sense of a mortality displacement (“harvesting effect”) because most existing studies focused on pandemic wave periods. An excess death in high-incidence intervals with respect to viral infections could also be associated with an inferior quality of treatment owing to excessive demands on health care services. Reduced availability of equipment and human resources or postponed therapeutic procedures are possible influencing factors. However, for a more precise statement, information on total mortality, including the death rate of outpatients, would be necessary because our data suggested at least no association with increased risk of absolute mortality within the inpatient environment in most disease groups. Third, data on total mortality would also be necessary to explore a possible shift of deaths from the inpatient to the outpatient setting during the pandemic, including nursing homes and hospices. Aside from conscious decisions to not admit all patients to a hospital in phases with high inpatient numbers because of the pandemic, the previously mentioned facts indicating patients’ reluctance to enter the inpatient health care facilities could be contributors to such a change in patients’ pathways. This could, in principle, influence all subgroups of diseases, but a distinct influence on neoplasms and cardiovascular diseases has to be assumed according to the above-mentioned studies, which may explain our findings in those *ICD-10* chapters.^13,31^ This, however, cannot be proven with our data set limited to inpatient cases and deserves further research but is supported by overall excess all-cause mortality for Germany when both inpatients and outpatients were included.^4^ Fourth, an actual reduction of in-hospital mortality could have occurred in some disease groups, which may be associated with behavioral changes regarding hygiene measures of patients, as well as health care personnel. Reduced incidences and corresponding absolute death counts are likely especially for the groups of infectious, respiratory, and digestive diseases; this likelihood is indicated by first examinations that showed an association with decreased rates of influenza, norovirus, and *Clostridium difficile*–associated infections.^35,36,37,38^ Moreover, nosocomial infections were reported to be diminished overall, which could lead to an improved inpatient outcome in all disease groups.^39,40^

### Limitations

This study has limitations. It was based on administrative data that were not stored for research interests but for remuneration reasons, which could affect the encoded information. Quality of the results depends to a large extent on the correct encoding of procedures and diagnoses at hospital discharge.^16^ This is particularly true for the encoding of SARS-CoV-2 infection because the specific *ICD-10* code was introduced April 1, 2020, and was retrospectively encoded thereafter for all previous cases. Therefore, misreporting or misclassification of SARS-CoV-2–related diseases and deaths could have occurred. However, regarding the main discharge diagnosis and the adequacy of hospitalization, as well as encoding, there is a continuous evaluation by reimbursement and health insurance companies that supports the assumption of overall valid information and also accounts for the supplemental information regarding the SARS-CoV-2 status as it is relevant for reimbursement. All analyses were performed on a case rather than patient level owing to data structure because neither cross-linking of patients between hospitals nor follow-up outside the investigated hospital network was possible.

To avoid selection bias, no specific inclusion criteria were applied but a broad spectrum of disease groups was examined. Because of the type of data, no specific causes of death could be determined and additional supporting information regarding patients’ specific medical history, imaging, laboratory results, medication, and treatment-related data was not available. Moreover, it was impossible to adjust the analyses for a number of potential confounders such as socioeconomic status owing to missing data. Because the investigation was retrospective, additional unknown factors may have influenced results. In addition, it was impossible to take into account the 30-day risk-adjusted mortality rates. The comparison of patient care metrics with data from 2019 harbors the possibility that observations were caused by year-dependent fluctuations. However, a comparison with previous years’ data has also been considered a valid method of comparison in several other studies investigating changes in health care use during the COVID-19 pandemic.^8,9,10,11,12,13,14,24,25,26^

## Conclusions

This cross-sectional analysis of a German multicenter inpatient database found association with both increased and decreased RMRs with respect to specific disease groups in 2020 compared with previous years. An association with increased absolute in-hospital mortality was observed only for the group of respiratory diseases but not for the overall cohort or other subgroups. After exclusion of patients with proven SARS-CoV-2 infection, no association with increased mortality but an association with decreased absolute mortality was found for the total cohort of 2020 and within several disease subgroups. Further research including the investigation of a potential shift of deaths to the outpatient setting is required.

## Notes

Supplement. **eTable 1.** *ICD-10* Disease Chapter **eTable 2.** *ICD-10* Codes Used to Calculate Elixhauser Comorbidity Score **eTable 3.** Relative Mortality Risks per *ICD-10* Chapter Adjusted for Age, Sex, and Elixhauser Comorbidity Score Comparing 2 Reference Periods (2016/2017, 2018/2019) With 2020 **eTable 4.** Relative Mortality Risks per *ICD-10* Chapter Adjusted for Age, Sex, Elixhauser Comorbidity Score, and SARS-CoV-2 Status Comparing 2 Reference Periods (2016/2017, 2018/2019) With 2020
