# Supplementary material for: A Comparative Analysis of In-Hospital Mortality per Disease Groups in Germany Before and During the COVID-19 Pandemic From 2016 to 2020
Source: JAMA Netw Open. 2022 Feb 15;5(2):e2148649. doi: 10.1001/jamanetworkopen.2021.48649 (PMC8848198; doi:10.1001/jamanetworkopen.2021.48649)
Supplement: Supplement. — eTable 1. ICD-10 Disease Chapter eTable 2. ICD-10 Codes Used to Calculate Elixhauser Comorbidity Score eTable 3. Relative Mortality Risks per ICD-10 Chapter Adjusted for Age, Sex, and Elixhauser Comorbidity Score Comparing 2 Reference Periods (2016/2017, 2018/2019) With 2020 eTable 4. Relative Mortality Risks per ICD-10 Chapter Adjusted for Age, Sex, Elixhauser Comorbidity Score, and SARS-CoV-2 Status Comparing 2 Reference Periods (2016/2017, 2018/2019) With 2020 [file jamanetwopen-e2148649-s001.pdf]

Supplemental Online Content

König S, Pellissier V, Hohenstein S, et al. A comparative analysis of in-hospital mortality per disease groups in Germany before and during the COVID-19 pandemic from 2016-2020. *JAMA Netw Open*. 2022;5(2):e2148649. doi:10.1001/jamanetworkopen.2021.48649

eTable 1. *ICD-10* Disease Chapter

eTable 2. *ICD-10* Codes Used to Calculate Elixhauser Comorbidity Score

eTable 3. Relative Mortality Risks per *ICD-10* Chapter Adjusted for Age, Sex, and Elixhauser Comorbidity Score Comparing 2 Reference Periods (2016/2017, 2018/2019) With 2020

eTable 4. Relative Mortality Risks per *ICD-10* Chapter Adjusted for Age, Sex, Elixhauser Comorbidity Score, and SARS-CoV-2 Status Comparing 2 Reference Periods (2016/2017, 2018/2019) With 2020

This supplemental material has been provided by the authors to give readers additional information about their work.

© 2022 König S et al. *JAMA Network Open.*

eTable 1. *ICD-10* Disease Chapter

ICD*-10 DISEASE CHAPTER	
Certain infectious and parasitic diseases	A00-B99
Neoplasms	C00-D48
Diseases of the blood and the blood-forming organs and certain disorders involving the immune system	D50-D90
Endocrine, nutritional and metabolic disorders	E00-E90
Mental, behavioral and neurodevelopmental disorders	F00-F99
Diseases of the nervous system	G00-G99
Diseases of the circulatory / cardiovascular system	I00-I99
Diseases of the respiratory system	J00-J99
Diseases of the digestive system	K00-K93
Diseases of the skin and subcutaneous tissue	L00-L99
Diseases of the musculoskeletal system and the connective tissue	M00-M99
Diseases of the genitourinary system	N00-N99
Others	P00-P96, Q00-Q99, R00-R99, S00-T98, U00-U99, V01-Y84, Z00-Z99

* International Statistical Classification of Diseases and Related Health Problems (ICD-10-GM [German Modification])

© 2022 König S et al. *JAMA Network Open.*

eTable 2. ***ICD-10*** **Codes Used to Calculate Elixhauser Comorbidity Score**

**ICD*-CODES USED TO CALCULATE ELIXHAUSER COMORBIDITY SCORE**		
Item	*Weight*	*ICD*-10-GM-code*
AIDS / HIV	0	B20, B21, B22, B23, B24
Alcohol Abuse	-1	F10, E52, G62.1, I42.6, K29.2, K70.0, K70.3, K70.9, T51, Z50.2, Z71.4, Z72.1
Blood Loss Anemia	-3	D50.0
Cardiac Arrhythmia	0	I44.1, I44.2, I44.3, I45.6, I47, I48, I49, R00.0, R00.1, R00.8, T82.1, Z45.00, Z45.01, Z95.0
Chronic Pulmonary Disease	3	I27.8, I27.9, J40, J41, J42, J43, J44, J45, J46, J47, J60, J61, J62, J63, J64, J65, J66, J67, J68.4, J70.1, J70.3
Chronic Renal Failure	6	I12.0, I31.1, N18, N19, N25.0, Z49.0, Z49.1, Z49.2, Z94.0, Z99.2
Coagulopathy	11	D65, D66, D67, D68, D69.1, D69.3, D69.4, D69.5, D69.6
Congestive Heart Failure	9	I09.0, I11.0, I13.0, I13.2, I25.5, I42.0, I42.1, I42.2, I42.5, I42.6, I42.7, I42.8, I42.9, I43, I50
Deficiency Anemia	-2	D50.8, D50.9, D51, D52, D53
Depression	-5	F20.4, F31.3 - F31.5, F32, F33, F34.1, F41.2, F43.2
Diabetes Mellitus, Uncomplicated	0	E10.0, E10.1, E10.9, E11.0, E11.1, E11.9, E12.0, E12.1, E12.9, E13.0, E13.1, E13.9, E14.0, E14.1, E14.9 (excluding E10.2, E10.3, E10.4, E10.5, E10.6, E10.7, E10.8, E11.2, E11.3, E11.4, E11.5, E11.6, E11.7, E11.8, E12.2, E12.3, E12.4, E12.5, E12.6, E12.7, E12.8, E13.2, E13.3, E13.4, E13.5, E13.6, E13.7, E13.8, E14.2, E14.3, E14.4, E14.5, E14.6, E14.7, E14.8)
Diabetes Mellitus, Complicated	-3	E10.2, E10.3, E10.4, E10.5, E10.6, E10.7, E10.8, E11.2, E11.3, E11.4, E11.5, E11.6, E11.7, E11.8, E12.2, E12.3, E12.4, E12.5, E12.6, E12.7, E12.8, E13.2, E13.3, E13.4, E13.5, E13.6, E13.7, E13.8, E14.2, E14.3, E14.4, E14.5, E14.6, E14.7, E14.8

© 2022 König S et al. *JAMA Network Open.*

Drug Abuse	**-7**	F11, F12, F13, F14, F15, F16, F18, F19, Z71.5, Z72.2
Fluid And Electrolyte Disorders	11	E22.2, E86, E87
Hypertension (combined uncomplicated and complicated)	-1	I10, I11, I12, I13, I15
Hypothyroidism	0	E00, E01, E02, E03, E89.0
Liver Disease	4	B18, I85, I86.4, I98.2, K70, K71.1, K71.3, K71.4, K71.5, K71.7, K72, K73, K74, K76.0, K76.2, K76.9, Z94.4
Lymphoma	6	C81, C82, C83, C84, C85, C88, C96, C90.0, C90.2
Metastatic Cancer	14	C77, C78, C79, C80
Neurological Disorders, other	5	G10, G11, G12, G13. G20, G21, G22, G25.4, G25.5, G31.2, G31.8, G31.9, G32, G35, G36, G37, G40, G41, G93.1, G93.4, R47.0, R56
Obesity	-5	E66
Paralysis	5	G04.1, G11.4, G80.1, G80.2, G81, G82, G83.0, G83.1, G83.2, G83.3, G83.4, G83.9
Peptic Ulcer Disease, Excluding Bleeding	0	K25.7, K25.9, K26.7, K26.9, K27.7, K27.9, K28.7, K28.9
Peripheral Vascular Disorders	3	I70, I71, I73.1, I73.8, I73.9, I77.1, I79.0, I79.2, Z95.81, Z95.88, Z95.9
Psychoses	-5	F20, F22, F23, F24, F25, F28, F29, F30.2, F31.2, F31.5
Pulmonary Circulation Disorder	6	I26, I27, I28.0, I28.8, I28.9
Rheumatoid Arthritis / Collagen Vascular Diseases	0	L94.0, L94.1, L94.3, M05, M06, M08, M12.0, M12.3, M30, M31.0, M31.1, M31.2, M31.3, M32, M33, M34, M35, M45, M46.1, M46.8, M46.9

© 2022 König S et al. *JAMA Network Open.*

Solid Tumor Without Metastases	**7**	C00, C01, C02, C03, C04, C05, C06, C07, C08, C09, C10, C11, C12, C13, C14, C15, C16, C17, C18, C19, C20, C21, C22, C23, C24, C25, C26, C30, C31, C32, C33, C34, C37, C38, C39, C40, C41, C43, C45, C46, C47, C48, C49, C50, C51, C52, C53, C54, C55, C56, C57, C58, C60, C61, C62, C63, C64, C65, C66, C67, C68, C69, C70, C71, C72, C73, C74, C75, C76, C97
Valvular Heart Disease	0	I05, I06, I07, I08, I09.1, I34, I35, I36, I37, I38, I39, Q23.0, Q23.1, Q23.2, Q23.3, Z95.2, Z95.3, Z95.4
Weight Loss	9	E40, E41, E42, E43, E44, E45, E46, R63.4, R64

* International Statistical Classification of Diseases and Related Health Problems (ICD-10-GM [German Modification])

© 2022 König S et al. *JAMA Network Open.*

eTable 3. Relative Mortality Risks per *ICD-10* Chapter Adjusted for Age, Sex and Elixhauser Comorbidity Score Comparing 2 Reference Periods (2016/2017, 2018/2019) With 2020

**Mortality rate (per 100,000)**			**RMR (95%CI)**			
2016-2017	2018-2019	2020	RMR 2016-17	P*	RMR 2018-19	P*
Total						
2,177	2,177	2,515	1.09 (1.07-1.10)	<**0.001**	1.10 (1.08-1.12)	<**0.001**
Certain infectious and parasitic diseases						
4,822	4,431	6,502	1.28 (1.21-1.35)	<**0.001**	1.36 (1.29-1.44)	<**0.001**
Diseases of the blood and blood-forming organs and certain disorders involving the immune mechanism						
1,849	1,664	1,954	1.05 (0.85-1.29)	0.666	1.13 (0.92-1.39)	0.253
Diseases of the circulatory system						
3,817	3,741	3,989	1.00 (0.97-1.03)	0.794	1.02 (0.99-1.05)	0.146
Diseases of the digestive system						
2,030	1,989	2,096	0.95 (0.90-1.00)	**0.045**	0.96 (0.91-1.01)	0.139
Diseases of the genitourinary system						
2,055	2,027	2,027	0.90 (0.84-0.96)	**0.002**	0.94 (0.87-1.00)	0.053
Diseases of the musculoskeletal system and connective tissue						
246	258	333	1.36 (1.18-1.56)	<**0.001**	1.24 (1.08-1.42)	**0.003**
Diseases of the nervous system						
989	1,180	1,303	1.25 (1.11-1.40)	<**0.001**	1.05 (0.93-1.18)	0.417
Diseases of the respiratory system						
3,960	4,115	6,892	1.54 (1.48-1.60)	<**0.001**	1.51 (1.45-1.57)	<**0.001**
Diseases of the skin and subcutaneous tissue						
696	760	772	1.07 (0.87-1.31)	0.544	0.98 (0.80-1.20)	0.874
Endocrine, nutritional and metabolic diseases						
2,338	2,369	2,408	0.96 (0.88-1.06)	0.461	1.00 (0.91-1.10)	0.961
Mental, Behavioral and Neurodevelopmental disorders						
398	439	461	1.16 (0.96-1.41)	0.127	1.07 (0.89-1.30)	0.473

© 2022 König S et al. *JAMA Network Open.*

Neoplasms						
4,656	4,532	4,584	0.98 (0.95-1.01)	0.262	1.01 (0.97-1.04)	0.644
Other						
1,072	1,085	1,195	1.05 (1.00-1.11)	**0.049**	1.06 (1.01-1.12)	**0.017**

* Significant p values are marked (broad)

RMR: Relative mortality risk; SARS-CoV-2: Severe acute respiratory syndrome coronavirus type 2

© 2022 König S et al. *JAMA Network Open.*

eTable 4. Relative Mortality Risks per *ICD-10* Chapter Adjusted for Age, Sex, Elixhauser Comorbidity Score and SARS-CoV-2 Status Comparing 2 Reference Periods (2016/2017, 2018/2019) With 2020

**Mortality rate (per 100,000)**			**RMR (95%CI)**			
2016-2017	2018-2019	2020	RMR 2016-17	P*	RMR 2018-19	P*
Total						
2,177	2,177	2,515	1.00 (0.98-1.01)	0.799	1.01 (0.99-1.02)	0.287
Certain infectious and parasitic diseases						
4,822	4,431	6,502	1.24 (1.17-1.31)	**<0.001**	1.32 (1.24-1.39)	**<0.001**
Diseases of the blood and blood-forming organs and certain disorders involving the immune mechanism						
1,849	1,664	1,954	0.99 (0.80-1.22)	0.938	1.07 (0.87-1.32)	0.531
Diseases of the circulatory system						
3,817	3,741	3,989	0.98 (0.95-1.01)	0.103	1.00 (0.97-1.03)	0.942
Diseases of the digestive system						
2,030	1,989	2,096	0.92 (0.88-0.97)	**0.003**	0.94 (0.89-0.99)	**0.014**
Diseases of the genitourinary system						
2,055	2,027	2,027	0.86 (0.81-0.93)	**<0.001**	0.90 (0.84-0.96)	**0.003**
Diseases of the musculoskeletal system and connective tissue						
246	258	333	1.25 (1.08-1.44)	**0.003**	1.14 (0.99-1.31)	0.079
Diseases of the nervous system						
989	1,180	1,303	1.21 (1.08-1.37)	**0.002**	1.02 (0.91-1.15)	0.748
Diseases of the respiratory system						
3,960	4,115	6,892	1.10 (1.05-1.16)	**<0.001**	1.08 (1.03-1.13)	**<0.001**
Diseases of the skin and subcutaneous tissue						
696	760	772	1.01 (0.82-1.25)	0.924	0.93 (0.76-1.15)	0.507
Endocrine, nutritional and metabolic diseases						
2,338	2,369	2,408	0.93 (0.84-1.02)	0.140	0.97 (0.88-1.06)	0.480
Mental, Behavioral and Neurodevelopmental disorders						
398	439	461	1.15 (0.95-1.40)	0.152	1.06 (0.88-1.29)	0.531

© 2022 König S et al. *JAMA Network Open.*

Neoplasms						
4,656	4,532	4,584	0.96 (0.93-1.00)	**0.025**	0.99 (0.96-1.02)	0.499
Other						
1,072	1,085	1,195	0.99 (0.94-1.04)	0.640	1.00 (0.95-1.05)	0.936

* Significant p values are marked (broad)

RMR: Relative mortality risk; SARS-CoV-2: Severe acute respiratory syndrome coronavirus type 2

© 2022 König S et al. *JAMA Network Open.*
